# Congestion–Perfusion Phenotypes and In-Hospital Mortality in Acute Heart Failure: Phenotype-Specific Prognostic Markers and Right-Heart Involvement

**DOI:** 10.3390/medicina62071422

**Published:** 2026-07-22

**Authors:** Mara Diaconu, Dan-Cristian Popescu, Diana Țînț, Alexandru-Cristian Nechita

**Affiliations:** 1 Department of Cardiology, Clinical Emergency Hospital Sfântul Pantelimon, 021659 Bucharest, Romania; mara2228@gmail.com (M.D.); popescu_dan_95@yahoo.com (D.-C.P.); cardionechita@yahoo.com (A.-C.N.); 2 Faculty of Medicine, Carol Davila University of Medicine and Pharmacy, 020021 Bucharest, Romania; 3 Faculty of Medicine, Transilvania University, 500036 Brasov, Romania; 4 Department of Cardiology, ICCO Clinics, 500059 Brasov, Romania

**Keywords:** acute heart failure, congestion, hypoperfusion, phenotypes, in-hospital mortality, echocardiography, right ventricular function, risk stratification

## Abstract

*Background and Objectives*: Acute heart failure (AHF) has heterogeneous clinical profiles and considerable short-term mortality. Bedside evaluation of congestion and peripheral perfusion may help identify clinically relevant risk groups. This study aimed to evaluate the relationship between a non-invasive congestion–perfusion classification and in-hospital mortality in patients hospitalized with AHF and to explore the prognostic relevance of clinical, biological, and right-heart variables within the phenotypes. *Materials and Methods*: We performed an observational study that analyzed 790 patients hospitalized with AHF. Patients were classified at presentation into four predefined congestion–perfusion phenotypes: non-congested/preserved perfusion, congested/preserved perfusion, non-congested/impaired perfusion, and congested/impaired perfusion. Congestion was defined using pulmonary and systemic markers, whereas impaired perfusion was defined by the presence of at least two predefined criteria of hypoperfusion. Clinical, biological, and echocardiographic parameters were analyzed according to phenotype and in-hospital mortality. Discriminative performance was evaluated using ROC curve analysis, and logistic regression models were constructed within the congestive phenotypes to investigate the prognostic value of combined clinical and biological markers, with bootstrap internal validation in the combined models. *Results*: 78 patients died during hospitalization, with different rates across phenotypes; 2.9% in non-congested/preserved perfusion, 3.0% in non-congested/impaired perfusion, 8.0% in congested/preserved perfusion, and 19.5% in congested/impaired perfusion. In congestive phenotypes, several adverse markers were identified. Exploratory combined models demonstrated discriminatory performance in the congested/preserved perfusion phenotype (AUC 0.731) and in the congested/impaired perfusion phenotype (AUC 0.838); bootstrap optimism-corrected AUCs were 0.693 and 0.785, respectively. Right ventricular parameters were more strongly associated with mortality in the congested/impaired perfusion phenotype. LVEF showed limited discrimination within individual phenotypes. *Conclusions*: The highest in-hospital mortality was observed in the congestive/impaired perfusion phenotype, with a more adverse clinical, biological, and echocardiographic profile. Within the congestive phenotypes, exploratory prognostic marker patterns were partially overlapping, while LVEF showed limited discrimination.

## 1. Introduction

Acute heart failure (AHF) is one of the most frequent causes of hospitalization among patients with cardiovascular disease and is associated with high short-term mortality [1,2]. Early stratification in AHF remains challenging despite advances in diagnostic and therapeutic strategies because patients present with heterogenous clinical profiles, different degrees of congestion, variable hemodynamic compromise, and multiple comorbidities [3,4,5].

Congestion is a central pathophysiological and clinical feature of AHF. However, it should not be regarded as a single uniform clinical entity. It can reflect different pathophysiological mechanisms, including fluid accumulation, increased cardiac filling pressures, fluid redistribution, and variable involvement of intravascular and extravascular compartments [6,7]. From a clinical perspective, pulmonary congestion, systemic venous congestion, and combined congestion may represent distinct patterns of hemodynamic burden, defining phenotypes with different responses to treatments or outcomes [8,9].

Peripheral hypoperfusion represents an important pathophysiological element of AHF severity, reflecting the inability of the cardiovascular system to maintain adequate oxygen delivery to meet metabolic demands [4]. When present alongside congestion, it is associated with an increased risk of adverse outcomes [10,11].

The combination of congestion and perfusion into clinically meaningful profiles originates from the hemodynamic subsets described by Forrester, which classified patients according to invasive measurements of pulmonary capillary pressure and cardiac index [12,13]. This invasive framework identified patients with high filling pressures, reduced cardiac output, or both, linking congestion and hypoperfusion to different degrees of hemodynamic severity [13]. This concept was subsequently translated into bedside clinical assessment through the “wet/dry“ and “warm/cold“ profiles, in which congestion and perfusion are evaluated using non-invasive clinical signs [14,15]. In recent cohorts of AHF patients, this clinical classification has helped identify phenotypes with different baseline characteristics, management strategies, and outcomes [16,17].

Echocardiography provides additional information that complements bedside clinical assessment in AHF. Although left ventricular ejection fraction (LVEF) is a central parameter for left ventricular (LV) dysfunction and for the classification of AHF, it does not fully characterize the complexity of acute decompensation and short-term risk [9]. Right ventricular (RV) parameters, such as tricuspid annular plane systolic excursion (TAPSE), Doppler Tissue Imaging-derived tricuspid lateral annular systolic velocity (S’), and pulmonary artery systolic pressure (PASP) have been associated with adverse outcomes in patients with AHF [18,19,20]. More recently, the TAPSE/PASP ratio has been described as a non-invasive marker of RV–pulmonary artery coupling and has been associated with increased mortality in patients with AHF [21,22].

This study aimed to evaluate the association between predefined, non-invasive congestion–perfusion phenotypes and all-cause in-hospital mortality in patients with AHF. More importantly, we sought to explore the patterns of association between clinical and biological parameters and mortality within the phenotypes and to assess the contribution of right ventricular dysfunction and LVEF to short-term risk stratification.

## 2. Materials and Methods

### 2.1. Study Population

This observational study with both retrospective and prospective components included 790 patients hospitalized with AHF in the Cardiology Department of the Clinical Emergency Hospital “Sf. Pantelimon“ Bucharest between January 2024 and May 2026. The diagnosis of AHF was established according to the 2021 European Society of Cardiology Guidelines for the diagnosis and treatment of acute and chronic heart failure (HF), based on clinical signs and symptoms of congestion, hypoperfusion, elevated NT-proBNP levels, and echocardiographic signs of cardiac abnormalities [23]. This study was performed in a hospital with a level II intensive cardiac care unit [24]. The primary endpoint of the study was all-cause in-hospital mortality.

The inclusion criteria consisted of complete data regarding clinical assessment of congestion, signs of hypoperfusion, arterial blood gas analysis, NT-proBNP measurement, and transthoracic echocardiography evaluation performed at presentation. Patients with missing data, as well as patients with acute coronary syndrome, myocarditis, or Takotsubo cardiomyopathy were excluded from the study population.

All study procedures were performed according to the Declaration of Helsinki. This study was approved by the Ethics Committee of Clinical Emergency Hospital “Sf. Pantelimon“ Bucharest.

### 2.2. Data Collection

For the retrospective component, the predefined variables were extracted from existing medical records. For the prospective component, the same predefined variables were systematically collected as part of routine assessment of patients with AHF. Patients underwent clinical evaluation, laboratory testing, and echocardiographic assessment at presentation in the ED. Data collected included demographic characteristics (age, sex), cardiovascular risk factors (hypertension, smoking status, obesity, dyslipidemia, and diabetes), and other relevant cardiovascular comorbidities (atrial fibrillation, prior diagnostic of HF, ischemic heart disease, chronic kidney disease, or chronic obstructive pulmonary disease). Clinical parameters collected at presentation included systolic blood pressure (SBP), diastolic blood pressure (DBP), heart rate (HR), peripheral oxygen saturation, signs of congestion, and hypoperfusion.

Laboratory parameters included NT-proBNP, hemoglobin, high-sensitivity cardiac troponin T (hs-cTnT), C-reactive protein (CRP), serum sodium, urea, creatinine, arterial lactate, and other routinely available variables.

Echocardiographic assessment was performed in the ED using a GE Logiq S7 XDclear 2.0 ultrasound system (GE Healthcare, Wauwatosa, WI, USA). Variables collected included LVEF, systolic and diastolic function of the LV and RV, LV filling pressures, and signs of pulmonary hypertension and congestion.

Treatment-related variables included intravenous (iv) furosemide administration during the initial management, inotropic support, non-invasive mechanical ventilation (NIMV), and invasive mechanical ventilation (IMV). Patients receiving chronic oral furosemide before admission were switched to iv furosemide during hospitalization, while patients in whom diuretic treatment was initiated in hospital initially received iv furosemide.

### 2.3. Definition of Clinical Congestion–Perfusion Phenotypes

Patients were classified into four groups using predefined, non-invasive clinical and paraclinical criteria based on the assessment of congestion and peripheral perfusion at presentation. The classification was predefined before the statistical analysis and outcome modeling and was applied uniformly to all eligible patients. The variables required for phenotype classification were systematically assessed and recorded as part of the routine evaluation of patients admitted with AHF. Subjective clinical signs were considered present only when explicitly documented in the admission assessment.

Congestion was evaluated using clinical and echocardiographic markers reflecting pulmonary and systemic overload and was defined as follows:

- Pulmonary congestion: presence of pulmonary rales or pleural effusion.
- Systemic congestion: presence of peripheral edema, jugular vein distention, hepatomegaly, ascites, or an inferior vena cava (IVC) diameter > 21 mm.
- Pulmonary and systemic congestion: presence of both systemic and pulmonary congestion.

Patients were considered congested if either pulmonary or systemic congestion was present.

Perfusion status was assessed using clinical and biological markers of circulatory impairment. Hypotension was defined as SBP < 90 mmHg. Impaired perfusion was defined as the presence of at least two of the following criteria:

- SBP < 90 mmHg.
- Arterial lactate > 2 mmol/L.
- Cold extremities.
- Oliguria.
- Mental confusion.
- Dizziness.
- Narrow pulse pressure.

Patients who did not meet at least two of these criteria were classified as having preserved perfusion.

The study population was divided into four clinical congestion–perfusion phenotypes:

- Non-congested/preserved perfusion.
- Congested/preserved perfusion.
- Non-congested/impaired perfusion.
- Congested/impaired perfusion.

### 2.4. Echocardiographic Assessment

Transthoracic echocardiography was performed at presentation in the ED by a cardiology specialist according to current recommendations for cardiac chamber quantification using the same ultrasound machine [25]. LVEF was calculated by Simpson’s biplane method. RV systolic function was assessed using TAPSE, measured by M-mode echocardiography in the apical four-chamber view and S’. Right-sided pressure parameters were derived from tricuspid regurgitation (TR) Doppler measurements. The right ventricular–right atrial systolic pressure gradient (*RV–RA gradient*) was calculated using the modified Bernoulli equation [26]:

$$RV-RA\;gradient=4\times {TRV}^{2}$$

where *TRV* represents peak TR velocity.

Estimated *PASP* was calculated using the formula

$$PASP=4\times {TRV}^{2}+estimated\;RAP$$

where *RAP* represents estimated right atrial pressure, from the size and collapsibility of the IVC [27].

The TAPSE/PASP ratio was used as a non-invasive index of RV–pulmonary artery coupling [28]. Lower values were considered to reflect poorer coupling between RV systolic function and pulmonary artery load.

### 2.5. Statistical Analysis

Continuous variables were reported as mean ± standard deviation or median with interquartile range (IQR), according to their distribution and the purpose of the analysis. Categorical variables were reported as relative frequency (%) and absolute frequency (No.). Differences between survivors and non-survivors were compared according to the primary endpoint of all-cause in-hospital mortality using the independent-samples *t*-test, the Mann–Whitney U test for continuous variables, the chi-square test, or Fisher’s exact test for categorical variables. Statistical significance was defined as a two-sided *p*-value < 0.05.

In-hospital mortality was first evaluated in the overall cohort and subsequently across the four clinical congestion–perfusion phenotypes. An additional multivariable logistic regression analysis was performed for the association between clinical phenotypes and in-hospital mortality. Given the limited number of events in the non-congested phenotypes, the adjusted analysis focused on the congested/impaired perfusion phenotype compared with all other phenotypes. The model was adjusted for prespecified baseline variables selected for clinical relevance: age, renal function, LVEF category, diabetes, ischemic etiology, and cardiogenic shock at presentation. A sensitivity analysis additionally included ICU admission, inotropic support, and non-invasive or invasive ventilation.

Phenotype-specific analyses were conducted to identify clinical, biological, and echocardiographic variables associated with in-hospital mortality. Owing to the small number of events in the non-congestive phenotypes, these analyses were considered exploratory. In the congestive phenotypes, the discriminative performance of individual variables associated with in-hospital mortality was further evaluated using the receiver operating characteristic (ROC) curve, calculating the area under the curve (AUC) with 95% confidence intervals (CIs) obtained by 1000 bootstrap resamples.

Exploratory multivariable logistic regression models were constructed separately for the congested/preserved perfusion and congested/impaired perfusion phenotypes using the adverse clinical and biological markers identified within each group. Model discrimination was assessed using ROC curve analysis and reported as apparent AUC, with bootstrap internal validation performed to estimate optimism-corrected AUC values.

Right ventricular parameters were analyzed in the two congestive phenotypes and compared between survivors and non-survivors. LVEF was analyzed across the four phenotypes as a marker of LV systolic function.

Treatment-related variables were compared across congestion–perfusion phenotypes using the chi-square test or Fisher’s exact test, as appropriate.

Statistical analyses were performed using IBM SPSS Statistics for Windows, version 26.0 (IBM Corp., Armonk, NY, USA).

## 3. Results

### 3.1. Baseline Characteristics

In total, 790 patients hospitalized with AHF were included in the analysis: 78 patients (9.9%) died during hospitalization, whereas 712 patients (90.1%) survived. Mean length of stay was 8.1 ± 4.9, with a median of 7 days. Baseline characteristics of the study population are summarized in Table 1. The mean age of the study population is 73.8 ± 12.2 years, non-survivors being significantly older than survivors (77.4 ± 9.9 vs. 73.4 ± 12.3, *p* = 0.001). The most frequent form of presentation was ADHF, observed in 609 patients (77.1%). APE was present in 153 patients (19.4%), while cardiogenic shock was identified in 28 patients (3.5%). Cardiogenic shock was more frequent among non-survivors compared with survivors (24.4% vs. 1.3%, *p* < 0.001).

Non-survivors more commonly presented with signs of congestion and hypoperfusion, including cold extremities (61.5% vs. 42.3%, *p* = 0.001), peripheral edema (59% vs. 47.1%, *p* = 0.045), pulmonary rales (69.2% vs. 51.8%, *p* = 0.003), and pleural effusion (42.3% vs. 20.1%, *p* < 0.001). Non-survivors presented with lower SBP (134.4 ± 32.3 vs. 147.0 ± 31.2 mmHg, *p* = 0.001), lower DBP (77.8 ± 18.3 vs. 85.2 ± 19.0 mmHg, *p* = 0.001), and lower oxygen saturation (91.8 ± 6.0 vs. 93.3 ± 5.3%, *p* = 0.044). Patients who died during hospitalization had higher levels of NT-proBNP (17,408 ± 13,007 vs. 8542 ± 11,521 pg/mL, *p* < 0.001), higher levels of hs-cTnT (93.9 ± 172.7 vs. 49.5 ± 76.4 pg/mL, *p* = 0.027), and higher lactate levels (3.02 ± 2.28 vs. 2.08 ± 1.25, *p* < 0.001).

Echocardiographic assessment at presentation demonstrated lower TAPSE values (16.6 ± 6.3 vs. 18.5 ± 5.1 mm, *p* = 0.003), higher TR velocity (3.1 ± 0.7 vs. 2.8 ± 0.6 m/s, *p* = 0.006), higher RV–RA gradient (36.6 ± 13.9 vs. 31.7 ± 11.8 mmHg, *p* = 0.003), and larger IVC diameters (19.1 ± 6.1 vs. 17.0 ± 5.5 mm, *p* = 0.003). LVEF did not differ significantly between survivors and non-survivors (40.4 ± 16.9 vs. 43.7 ± 14.5%, *p* = 0.108).

### 3.2. Congestion–Perfusion Phenotype Distribution and In-Hospital Mortality

All patients were categorized into four congestion–perfusion phenotypes according to congestion and perfusion status (Table 2). In total, 137 patients (17.3%) were classified as non-congested with preserved perfusion, 415 (52.5%) patients as congested with preserved perfusion, 33 patients (4.2%) as non-congested with impaired perfusion, and 205 patients (25.9%) as congested with impaired perfusion. In-hospital mortality differed significantly across the four phenotypes (*p* < 0.001).

**Figure 1:**
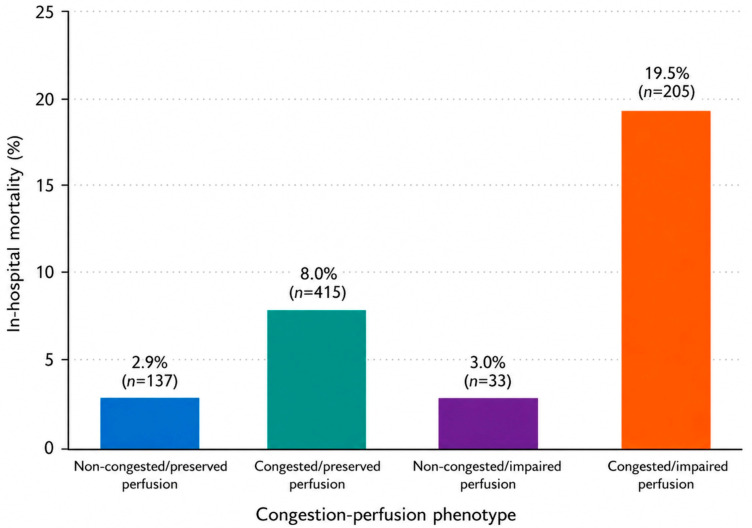
In-hospital mortality according to congestion–perfusion phenotype.

The lowest mortality rate was observed among non-congested with preserved perfusion (2.9%), whereas the highest mortality rate was observed in patients with congestion and impaired perfusion (19.5%). Patients with congestion and preserved perfusion had an intermediate mortality rate of 8.0%. In the non-congested/impaired perfusion phenotype, only one in-hospital death was recorded. Therefore, the observed mortality rate in this subgroup is reported descriptively and should not be interpreted comparatively (Figure 1).

In the adjusted logistical regression analysis, the congested/impaired perfusion phenotype remained associated with in-hospital mortality compared with all other phenotypes after adjustment for age, renal function, LVEF category, diabetes, ischemic etiology, and cardiogenic shock at presentation (adjusted OR 2.79, 95% CI 1.63–4.76, *p* < 0.001). In a sensitivity analysis additionally including ICU admission, inotropic support and non-invasive or invasive ventilation, this association remained significant (adjusted OR 2.50, 95% CI 1.22–5.12, *p* = 0.012).

### 3.3. Phenotype-Specific Adverse Prognostic Markers

Phenotype-specific adverse prognostic markers were assessed by comparing survivors and non-survivors within each clinical phenotype (Table 3). Given the low number of deaths in the non-congested phenotypes, results in these subgroups were interpreted cautiously.

In the non-congested/preserved perfusion phenotype, only four in-hospital deaths were recorded. Non-survivors had higher NT-proBNP levels (16,355 [10,908–30,451] vs. 2388 [1286–4640] pg/mL, *p* = 0.02) and lower hemoglobin concentrations (11.4 [10.1–12.1] vs. 13.1 [11.7–14.6] g/dL, *p* = 0.05). In the non-congested/impaired perfusion phenotype, only one in-hospital death was recorded. Therefore, no reliable conclusions were drawn from this subgroup.

**Figure 2:**
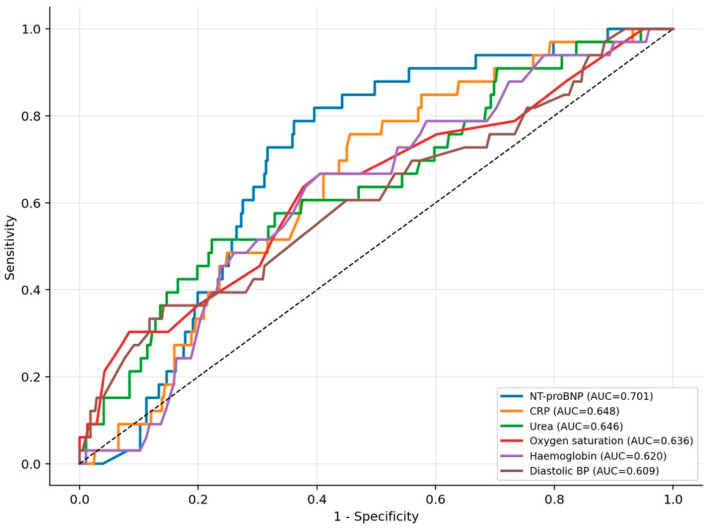
ROC curves of individual parameters for in-hospital mortality in the congested/preserved perfusion phenotype.

Among patients with congestion and preserved perfusion, in-hospital death was associated with higher NT-proBNP levels (10,767 [7606–16,065] vs. 4932 [2317–10,986] pg/mL, *p* < 0.001), higher CRP levels (18.8 [10.8–39.1] vs. 8.79 [3.01–23.5] mg/dL, *p* = 0.005), and higher urea levels (71.8 [45.8–99.7] vs. 53.2 [39.3–72.4] mg/dL, *p* = 0.009). Non-survivors also had lower hemoglobin levels (11.5 [10.6–13.0] vs. 12.7 [11.2–14.3] g/dL, *p* = 0.024), lower oxygen saturation (92 [87–95] vs. 95 [90–97]%, *p* = 0.037), and lower DBP (80 [61–94] vs. 83 [73.2–98] mmHg, *p* = 0.037). In this phenotype, individual clinical and biological markers showed moderate discrimination for in-hospital mortality (Figure 2). NT-proBNP had the highest discriminatory performance (AUC 0.701, 95% CI 0.626–0.772), followed by CRP (AUC 0.648, 95% CI 0.564–0.729), urea (AUC 0.646, 95% CI 0.554–0.749), oxygen saturation (AUC 0.636, 95% CI 0.528–0.742), hemoglobin (AUC 0.620, 95% CI 0.525–0.709), and DBP (AUC 0.609, 95% CI 0.503–0.718) (Table 4).

**Figure 3:**
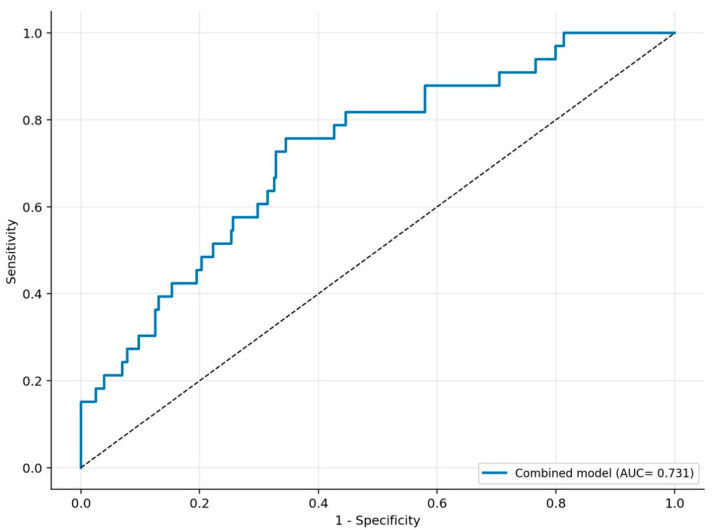
ROC curve of the exploratory combined clinical–biological model for in-hospital mortality in the congested/preserved perfusion phenotype.

An exploratory combined clinical–biological model, including NT-proBNP, CRP, urea, hemoglobin, oxygen saturation, and DBP achieved an AUC of 0.731 (95% CI 0.643–0.815) (Figure 3). Bootstrap internal validation yielded an optimism-corrected AUC of 0.693. Given the limited number of events, this combined model should be interpreted as exploratory and hypothesis-generating rather than as a validated predictive model.

The most extensive adverse prognostic profile was identified in patients with combined congestion and impaired perfusion. Within this phenotype, non-survivors had significantly higher NT-proBNP concentrations (19,451 [11,462–30,000] vs. 6144 [2769–12,564] pg/mL, *p* < 0.001), higher urea levels (90 [65.1–124] vs. 52.6 [39.3–79.2] mg/dL, *p* < 0.001), and higher creatinine concentrations (1.73 [1.24–2.18] vs. 1.07 [1.00–1.62] mg/dL, *p* < 0.001). This phenotype was also characterized by lower SBP (126 [101–143] vs. 147 [121–170] mmHg, *p* = 0.001), lower DBP (71.5 [65.5–90] vs. 85 [74–98] mmHg, *p* = 0.014), higher hs-cTnT levels (62.8 [26.6–97.6] vs. 31.4 [22.1–52.5], *p* = 0.003), higher CRP levels (18.1 [8.4–35.2] vs. 10.5 [4.14–26.4] mg/dL, *p* = 0.018), and higher lactate concentrations (3.02 [2.61–3.64] vs. 2.67 [2.26–3.40], *p* = 0.019). Furthermore, non-survivors had lower sodium concentrations (137 [131–141] vs. 139 [135–142] mmol/L, *p* = 0.021) and lower hemoglobin levels (11.9 [9.95–13.5] vs. 13.0 [11.5–14.2] g/dL, *p* = 0.043).

**Figure 4:**
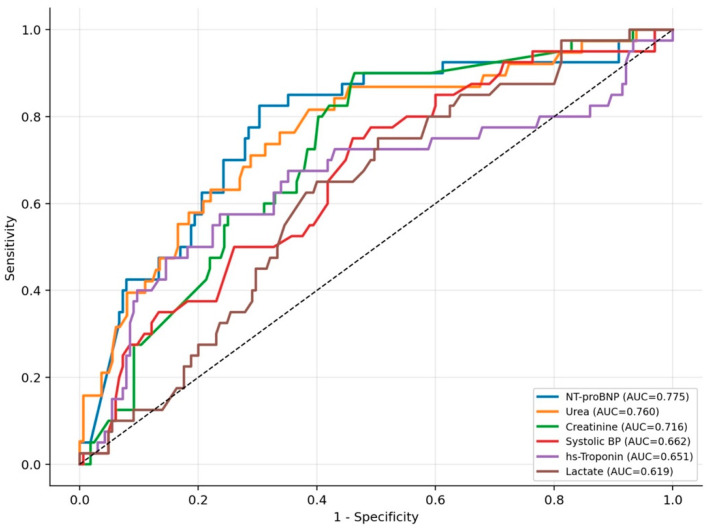
ROC curves of individual parameters for in-hospital mortality in the congested/impaired perfusion phenotype.

In this phenotype, individual parameters showed stronger discriminatory performance for in-hospital mortality than in the congested/preserved perfusion phenotype (Figure 4). NT-proBNP and urea demonstrated the highest individual AUC values: 0.775 (95% CI 0.687–0.853) and 0.760 (95% CI 0.669–0.844), respectively. Creatinine also showed good discriminatory performance, with an AUC of 0.716 (95% CI 0.634–0.790), whereas other markers showed more modest discrimination, including SBP (AUC 0.662, 95% CI 0.572–0.751), hs-cTnT (AUC 0.651, 95% CI 0.544–0.759), DBP (AUC 0.625, 95% CI 0.528–0.721), CRP (AUC 0.620, 95% CI 0.529–0.709), lactate (AUC 0.619, 95% CI 0.528–0.707), sodium (AUC 0.618, 95% CI 0.512–0.719), and hemoglobin (AUC 0.606, 95% CI 0.502–0.705) (Table 5).

**Figure 5:**
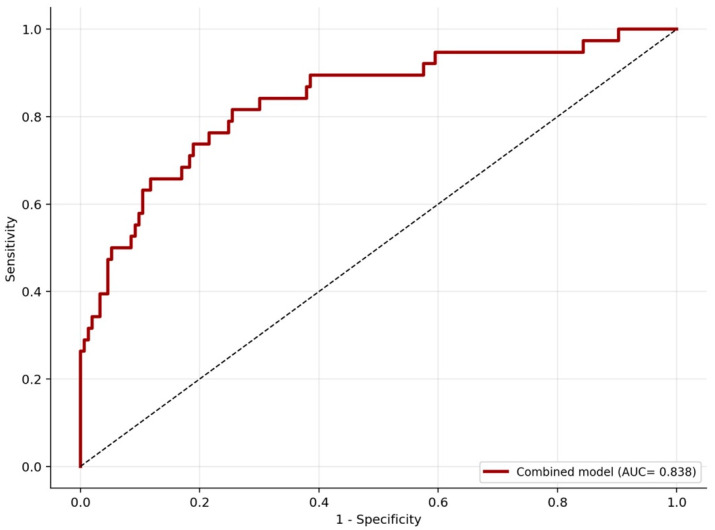
ROC curve of the exploratory combined clinical–biological model for in-hospital mortality in the congested/impaired perfusion phenotype.

An exploratory clinical–biological logistic model incorporating NT-proBNP, urea, creatinine, SBP, hs-cTnT, DBP, CRP, lactate, sodium, and hemoglobin achieved the highest discriminatory performance among evaluated models, with an AUC of 0.838 (95% CI 0.755–0.909) (Figure 5). Bootstrap internal validation yielded an optimism-corrected AUC of 0.785. Given the limited number of events, this combined model should be interpreted as exploratory and hypothesis-generating rather than as a validated predictive model.

### 3.4. Right Ventricular Dysfunction in Congestive Phenotypes

In the congested/preserved perfusion phenotype, right ventricular parameters showed a modest association with in-hospital mortality (Table 6). Non-survivors had a larger IVC diameter (20 [15–22] vs. 18 [13–21] mm, *p* = 0.042) and a higher RV–RA gradient (36 [29–43] vs. 31 [25–38] mmHg, *p* = 0.049).

In the congested/impaired perfusion phenotype, several right ventricular parameters differed significantly according to in-hospital outcome (Table 7). Non-survivors had lower TAPSE values compared with survivors (15.5 [12.8–18] vs. 18 [14–21] mm, *p* = 0.011) and lower RV S’ velocity (8 [7–9.6] vs. 11 [8.93–13] cm/s, *p* = 0.004). Patients who died during hospitalization had higher RV–RA gradient (34.5 [27–42] vs. 30 [22–39] mmHg, *p* = 0.027), higher estimated PASP (46.6 [36.4–63.8] vs. 39 [28–52] mmHg, *p* = 0.027), and significantly lower TAPSE/PASP (0.32 [0.22–0.47] vs. 0.46 [0.28–0.65], *p* = 0.006). IVC diameter was also larger in non-survivors (20 [17.8–24] vs. 18 [14–22] mm, *p* = 0.029), whereas TR velocity did not differ significantly between groups.

### 3.5. Left Ventricular Ejection Fraction Across Phenotypes

LVEF differed significantly across phenotypes (*p* < 0.001) (Table 8). The highest LVEF values were observed in the non-congested/preserved perfusion phenotype (53 [40–60]%), whereas the lowest were observed in patients with combined congestion and impaired perfusion (38 [27–50]%). However, within each phenotype, LVEF did not differ significantly between survivors and non-survivors. In the non-congested/preserved perfusion phenotype, LVEF was 54 [42–60]% in survivors and 42 [29.3–52.5]% in non-survivors, *p* = 0.235. In patients with congestion and preserved perfusion, LVEF was similar between survivors and non-survivors (42.5 [31.3–55]% vs. 45 [33–55]%, *p* = 0.335). Similarly, no significant differences were observed in the non-congested/impaired perfusion phenotype (46.5 [31–55]% vs. 29 [29–29]%, *p* = 0.430) or in the congested/impaired perfusion phenotype (39 [28–50]% vs. 35 [20–50]%, *p* = 0.236).

### 3.6. Treatment According to Congestion–Perfusion Phenotypes

Acute treatment differed across congestion–perfusion phenotypes (Table 9). Iv furosemide was administered to 734 patients (92.9%), inotropic support to 292 patients (11.6%), NIMV to 102 patients (12.9%), and IMV to 47 patients (5.9%). Patients in the congested/impaired perfusion phenotype required the greatest use of inotropic support, NIMV, and IMV in 21.0%, 19.5%, and 10.7% of cases, respectively. Differences across phenotypes were significant for iv furosemide (*p* < 0.001), inotropic support (*p* < 0.001), NIMV (*p* < 0.001), and IMV (*p* = 0.005).

## 4. Discussion

### 4.1. Principal Findings

This study evaluated a predefined, non-invasive congestion–perfusion classification in patients hospitalized with AHF and explored the prognostic relevance of clinical, biological, and echocardiographic variables across phenotypes. The observed mortality gradient across phenotypes was consistent with previous evidence, with the highest mortality occurring in the congested/impaired perfusion phenotype. Within the two congestive phenotypes, several clinical and biological variables remained independently associated with in-hospital mortality in exploratory multivariable analyses. These variables formed partially overlapping prognostic patterns, with NT-proBNP, urea, hemoglobin, DBP, and CRP contributing across both phenotypes. RV echocardiographic parameters showed clinically relevant associations with mortality, whereas LVEF demonstrated limited discrimination between survivors and non-survivors within individual phenotypes.

### 4.2. Congestion–Perfusion Phenotypes and Short-Term Risk

The four congestion–perfusion phenotypes showed different in-hospital mortality rates, with the lowest observed in patients without congestion and with preserved perfusion, and the highest mortality in those with concomitant congestion and impaired perfusion. Importantly, this association persisted after adjustment for key baseline clinical variables and remain significant in a sensitivity analysis additionally accounting for ICU admission, inotropic support, and ventilation. The non-congestive/impaired perfusion phenotype was the least frequent in this cohort, a finding consistent with previous clinical profile studies in AHF, in which “cold/dry“ presentations were uncommon [16,17]. Overall, this established mortality gradient provided the clinical framework for the subsequent within-phenotype analysis.

### 4.3. Clinical Interpretation of Phenotype-Specific Adverse Prognostic Markers

A key finding in the present study was the identification of clinical and biological markers associated with in-hospital mortality within each of the two congestive phenotypes. Although each phenotype showed a particular pattern of adverse prognostic markers, these patterns were partially overlapping. NT-proBNP, urea, hemoglobin, DBP, and CRP were associated with mortality in both phenotypes, suggesting a shared contribution of neurohormonal activation, renal dysfunction, inflammation, and systemic disease severity. In the congested/preserved perfusion phenotype, additional associations were observed for impaired oxygenation. In the congested/impaired perfusion phenotype, mortality was associated with a broader pattern additionally involving hs-cTnT, lactate, creatinine, lower SBP, and lower serum sodium. Therefore, these findings indicate partially overlapping patterns of prognostic markers rather than completely distinct sets of markers. In both congestive phenotypes, combined exploratory clinical–biological models showed higher apparent discrimination than individual markers, particularly in the congested/impaired perfusion phenotype. However, given the limited number of events and the reduction in AUC after bootstrap internal validation, these findings should be interpreted as hypothesis-generating rather than as validated predictive models. Overall, they support the possibility that short-term risk in AHF may be influenced by the cumulative effect of multiple adverse pathophysiological mechanisms rather than isolated parameters alone.

### 4.4. RV Echocardiographic Parameters Across Congestion–Perfusion Phenotypes

Another important finding of the present study relates to the prognostic role of RV echocardiographic parameters. Associations with in-hospital mortality were predominantly observed in patients with congestion and impaired perfusion, whereas fewer RV echocardiographic predictors emerged in the congested/preserved perfusion phenotype. In the congested/impaired perfusion phenotype, non-survivors had lower TAPSE and RV S’ velocity, with higher estimated PASP, higher RV–RA gradient, larger IVC diameter, and lower TAPSE/PASP ratio. These results suggest that adverse outcomes in this subgroup were associated not only with elevated systemic and pulmonary venous pressures but also with impaired RV systolic function and reduced RV–pulmonary arterial coupling. In contrast, in patients with congestion and preserved perfusion, prognostic echocardiographic parameters were limited to IVC diameter and RV–RA gradient, while other RV parameters showed limited discriminatory value. These findings support the growing evidence that right-heart involvement contributes substantially to risk stratification in AHF [29,30,31].

### 4.5. LVEF and Phenotype-Based Risk Assessment

LVEF remains fundamental for HF classification, but its role in short-term risk stratification during acute decompensation is limited. In this study, LVEF is different across phenotypes, with lower values in the congested/impaired perfusion phenotype and higher values in the non-congested/preserved perfusion phenotype. However, within individual phenotypes, LVEF did not consistently discriminate between survivors and non-survivors, suggesting that LV systolic function alone may not fully capture short-term risk when congestion and perfusion status are considered. This limited discriminatory value may reflect the fact that LVEF represents only one dimension of cardiac function and does not fully account for acute hemodynamic and systemic abnormalities associated with adverse outcomes. Within the congestion–perfusion phenotypes, short-term prognosis may be more closely related to congestion burden, peripheral hypoperfusion, right-heart dysfunction, myocardial injury, inflammation, renal impairment, and cardiometabolic stress. These findings support a multidimensional assessment extending beyond LV systolic function, consistent with the recent literature highlighting cardiometabolic modulation and broader mechanisms involved in HF progression [32].

### 4.6. Clinical Implications

The present findings suggest that phenotyping patients based on congestion and perfusion status may facilitate a more targeted evaluation of patients presenting with AHF. In patients with congestion and preserved perfusion, greater emphasis may be placed on markers of congestion severity, renal function, oxygenation, and neurohormonal activation, as these variables showed the strongest association with in-hospital mortality. Conversely, in patients with both congestion and impaired perfusion, risk assessment should include a careful evaluation of blood pressure, lactate, renal dysfunction, myocardial injury, and RV performance, as these parameters were more closely linked to in-hospital mortality. Therefore, congestion–perfusion profiling may refine early risk assessment by identifying not only higher-risk patients but also phenotype-specific patterns that should guide closer evaluation. Treatment intensity also paralleled the severity of the presenting phenotype, with greater use of inotropic support, NIMV, and IMV among patients with congestion and impaired perfusion. These findings suggest that early congestion–perfusion assessment may help identify patients likely to require more intensive hemodynamic and respiratory support. However, these interventions should be interpreted primarily as responses to greater acute clinical severity rather than as independent determinants of mortality.

### 4.7. Incremental Contribution and Future Directions

Although the mortality gradient across congestion–perfusion phenotypes is consistent with previous evidence, the incremental contribution of the present study lies in the evaluation of clinical, biological, and right-heart parameters associated with mortality within congestive phenotypes. Several variables remained independently associated with in-hospital mortality in the exploratory multivariable analysis. However, these variables formed partially overlapping rather than completely distinct prognostic patterns, with NT-proBNP, urea, hemoglobin, DBP, and CRP contributing across both phenotypes, while other markers showed phenotype-dependent associations.

Future studies should prospectively validate these within-phenotype associations in larger, multicenter cohorts using standardized assessments of congestion and perfusion. Serial evaluation of biomarkers reflecting congestion, myocardial injury, renal dysfunction, inflammation, cardiometabolic stress, and anemia should be explored. Integrating biomarkers with right-heart assessment and treatment response may improve risk stratification and determine whether this approach provides prognostic information beyond conventional clinical variables.

### 4.8. Strengths and Limitations

The present study has several strengths. Unlike previous investigations that focused primarily on clinical outcomes across congestion–perfusion phenotypes, the present study identified and characterized the factors associated with in-hospital mortality within individual phenotypes. The integration of RV echocardiographic parameters, such as indicators of RV function and RV–pulmonary arterial coupling, offers additional insight into the hemodynamic mechanisms contributing to adverse outcomes. Finally, the use of routinely available clinical, biological, and echocardiographic variables enhances the potential clinical applicability of the proposed approach.

Several limitations should be acknowledged. First, this was a single-center observational study, which may limit the generalizability of the findings. Second, the congestion–perfusion classification was based on predefined clinical, biological, and echocardiographic criteria rather than invasive hemodynamic measurement. While this approach reflects routine clinical practice and facilitates bedside applicability, we cannot exclude a certain degree of misclassification. Formal interobserver variability assessment for subjective clinical signs was not available as these signs were extracted from clinical documentation. Third, the relatively low number of events within certain phenotypes, particularly the non-congested/impaired perfusion subgroup, reduced statistical power for the subgroup analyses. Therefore, comparisons involving this phenotype should be regarded as descriptive. Fourth, although bootstrap internal validation was performed, the combined exploratory models were developed and evaluated within the same cohort, and the limited number of events may still have increased the risk of overfitting. Therefore, these models require external validation in independent cohorts. Finally, although adjusted and sensitivity analyses were performed for the association between clinical phenotypes and in-hospital mortality, residual confounding cannot be excluded. Detailed data on treatment timing, doses, diuretic response, vasodilators, and mechanical circulatory support were not systematically available, and residual treatment-related confounding cannot be excluded.

## 5. Conclusions

In patients hospitalized with AHF, a predefined non-invasive classification based on congestion and perfusion status identified clinically distinct phenotypes with different in-hospital mortality rates. The congested/impaired perfusion phenotype had the highest observed mortality and showed a broader adverse clinical, biological, and right-heart profile. Within the congestive phenotypes, several clinical, biological, and right-heart parameters showed exploratory phenotype-specific associations with in-hospital mortality, forming partially overlapping prognostic patterns, whereas LVEF demonstrated limited discrimination within individual phenotypes.

The present findings support viewing AHF as a heterogenous syndrome, in which congestion–perfusion phenotypes may help distinguish clinically relevant risk profiles, with different clinical, biological, and echocardiographic variables associated with in-hospital mortality.

## Figures and Tables

**Table 1:** Baseline characteristics of patients with AHF according to in-hospital outcome.

Variable	Patients (*N* = 790)	Survivors (*N* = 712)	Non-Survivors (*N* = 78)	*p*-Value
Age, years	73.8 ± 12.2	73.4 ± 12.3	77.4 ± 9.9	0.001
Male, %	48.7	48.4	50.6	0.748
Length of hospital stay, days	8.1 ± 4.9	7.8 ± 4.1	10.8 ± 9.0	0.004
Hypertension, %	91.9	92.7	84.6	0.013
Diabetes mellitus, %	42.7	41.9	50	0.167
Dyslipidemia, %	88.1	89.7	73.1	<0.001
Obesity, %	41.1	41.8	34.2	0.171
Smoking, %	25.2	25.5	22.1	0.467
Atrial fibrillation/Atrial flutter, %	59.7	59.3	64.1	0.409
ADHF, %	77.1	78.7	62.8	0.002
APE, %	19.4	20.1	12.8	0.123
Cardiogenic shock, %	3.5	1.3	24.4	<0.001
Cold extremities, %	44.2	42.3	61.5	0.001
Peripheral edema, %	48.2	47.1	59	0.045
Pulmonary rales, %	53.5	51.8	69.2	0.003
Pleural effusion, %	22.3	20.2	41	<0.001
SBP, mmHg	145.8 ± 31.6	147.0 ± 31.2	134.4 ± 32.3	0.001
DBP, mmHg	84.5 ± 19.0	85.2 ± 19.0	77.8 ± 18.3	0.001
Heart rate, bpm	99.1 ± 29.5	98.9 ± 29.7	101.0 ± 27.8	0.513
Oxygen saturation, %	93.2 ± 5.4	93.3 ± 5.3	91.8 ± 6.0	0.044
NT-proBNP, pg/mL	9417 ± 11,963	8542 ± 11,521	17,408 ± 13,007	<0.001
hs-cTnT, pg/mL	53.8 ± 91.4	49.5 ± 76.4	93.9 ± 172.7	0.027
Lactate, mmol/L	2.17 ± 1.41	2.08 ± 1.25	3.02 ± 2.28	<0.001
Sodium, mmol/L	138.0 ± 5.2	138.2 ± 5.0	136.4 ± 6.2	0.005
eGFR, ml/min/1.73 m^2^	62.5 ± 24.2	63.5 ± 24.2	52.7 ± 22.9	0.048
Hemoglobin, g/dL	12.6 ± 2.4	12.7 ± 2.4	11.6 ± 2.4	<0.001
CRP, mg/L	23.5 ± 40.8	21.9 ± 39.0	37.3 ± 53.4	0.015
LVEF, %	43.4 ± 14.7	43.7 ± 14.5	40.4 ± 16.9	0.108
VTI LVOT, cm	17.6 ± 5.4	17.7 ± 5.3	16.6 ± 6.3	0.162
TAPSE, mm	18.3 ± 5.1	18.5 ± 5.1	16.6 ± 6.3	0.003
TR velocity, m/s	2.9 ± 0.6	2.8 ± 0.6	3.1 ± 0.7	0.006
RV–RA gradient, mmHg	32.1 ± 12.2	31.7 ± 11.8	36.6 ± 13.9	0.003
IVC diameter, mm	17.2 ± 5.6	17.0 ± 5.5	19.1 ± 6.1	0.004

ADHF, acute decompensated heart failure; APE, acute pulmonary edema; SBP, systolic blood pressure; DBP, diastolic blood pressure; NT-proBNP, N-terminal prohormone of brain natriuretic peptide; hs-cTnT, high-sensitivity cardiac Troponin T; eGFR, estimated glomerular filtration rate; CRP, C-reactive protein; LVEF, left ventricular ejection fraction; VTI LVOT, velocity–time integral in the left ventricular outflow ejection tract; TAPSE, tricuspid annular plane systolic excursion; TR velocity, tricuspid regurgitation; RV–RA gradient, right ventricle–right atrium gradient; IVC diameter, inferior vena cava diameter.

**Table 2:** In-hospital outcomes according to congestion–perfusion phenotype.

Phenotype	*N*	Survivors	Non-Survivors	In-Hospital Mortality
Non-congested/preserved perfusion	137	133	4	2.9%
Congested/preserved perfusion	415	382	33	8.0%
Non-congested/impaired perfusion	33	32	1	3.0%
Congested/impaired perfusion	205	165	40	19.5%

**Table 3:** Phenotype-specific adverse prognostic markers associated with in-hospital mortality.

Phenotype	Adverse Marker	Survivors	Non-Survivors	*p*-Value
Non-congested/preserved perfusion	NT-proBNP, pg/mL	2388 [1286–4640]	16,355 [10,908–30,451]	0.02
	Hemoglobin, g/dL	13.1 [11.7–14.6]	11.4 [10.1–12.1]	0.05
Congested/preserved perfusion	NT-proBNP, pg/mL	4932 [2317–10,986]	10,767 [7606–16,065]	<0.001
	CRP, mg/dL	8.79 [3.01–23.5]	18.8 [10.8–39.1]	0.005
	Blood urea, mg/dL	53.2 [39.3–72.4]	71.8 [45.8–99.7]	0.009
	Hemoglobin, g/dL	12.7 [11.2–14.3]	11.5 [10.6–13.0]	0.024
	Oxygen saturation, %	95 [90–97]	92 [87–95]	0.037
	DBP, mmHg	83 [73.2–98]	80 [61–94]	0.037
Non-congested/impaired perfusion	-	-	-	
Congested/impaired perfusion	NT-proBNP, pg/mL	6144 [2769–12,564]	19,451 [11,462–30,000]	<0.001
	Blood urea, mg/dL	52.6 [39.3–79.2]	90 [65.1–124]	<0.001
	Creatinine, mg/dL	1.07 [1.00–1.62]	1.73 [1.24–2.18]	<0.001
	SBP, mmHg	147 [121–170]	126 [101–143]	0.001
	hs-cTnT pg/mL	31.4 [22.1–52.5]	62.8 [26.6–97.6]	0.003
	DBP, mmHg	85 [74–98]	71.5 [65.5–90]	0.014
	CRP, mg/dL	10.5 [4.14–26.4]	18.1 [8.4–35.2]	0.018
	Lactate, mmol/L	2.67 [2.26–3.40]	3.02 [2.61–3.64]	0.019
	Sodium, mmol/L	139 [135–142]	137 [131–141]	0.021
	Hemoglobin, g/dL	13.0 [11.5–14.2]	11.9 [9.95–13.5]	0.043

NT-proBNP, N-terminal prohormone of brain natriuretic peptide; CRP, C-reactive protein; DBP, diastolic blood pressure; SBP, systolic blood pressure; hs-cTnT, high-sensitivity cardiac Troponin T.

**Table 4:** ROC analysis for in-hospital mortality in the congested/preserved perfusion phenotype.

Marker	AUC	95% CI
NT-proBNP	0.701	0.626–0.772
CRP	0.648	0.564–0.729
Urea	0.646	0.554–0.749
Oxygen saturation	0.636	0.528–0.742
Hemoglobin	0.620	0.525–0.709
DBP	0.609	0.503–0.718
Exploratory combined model	0.731	0.643–0.815

NT-proBNP, N-terminal prohormone of brain natriuretic peptide; CRP, C-reactive protein; DBP, diastolic blood pressure.

**Table 5:** ROC analysis for in-hospital mortality in the congested/impaired perfusion phenotype.

Marker	AUC	95% CI
NT-proBNP	0.775	0.687–0.853
Urea	0.760	0.669–0.844
Creatinine	0.716	0.634–0.790
SBP	0.662	0.572–0.751
hs-cTnT	0.651	0.544–0.759
DBP	0.625	0.528–0.721
CRP	0.621	0.529–0.709
Lactate	0.619	0.528–0.707
Sodium	0.618	0.512–0.719
Hemoglobin	0.606	0.502–0.705
Exploratory combined model	0.838	0.755–0.909

NT-proBNP, N-terminal prohormone of brain natriuretic peptide; SBP, systolic blood pressure; hs-cTnT, high-sensitivity cardiac Troponin T; DBP, diastolic blood pressure; CRP, C-reactive protein.

**Table 6:** Right ventricular parameters in congested/preserved perfusion phenotype.

Parameter	Survivors	Non-Survivors	*p*-Value
TAPSE, mm	18 [15–22]	15 [13–19]	0.051
RV S’, cm/s	10 [8–12]	11 [9–12]	0.460
TR velocity, m/s	2.9 [2.5–3.4]	3.08 [2.5–3.5]	0.173
RV–RA gradient, mmHg	31 [25–38]	36 [29–43]	0.049
Estimated PASP, mmHg	39 [30–52]	42 [31.1–61.6]	0.127
TAPSE/PASP	0.49 [0.3–0.66]	0.38 [0.23–0.58]	0.107
IVC diameter, mm	18 [13–21]	20 [15–22]	0.042

TAPSE, tricuspid annular plane systolic excursion; RV S’, peak systolic tricuspid annular velocity measured by tissue Doppler imaging; TR velocity, tricuspid regurgitation; RV–RA gradient, right ventricle–right atrium gradient; PASP, pulmonary artery systolic pressure; IVC diameter, inferior vena cava diameter.

**Table 7:** Right ventricular parameters in congested/impaired perfusion phenotype.

Parameter	Survivors	Non-Survivors	*p*-Value
TAPSE, mm	18 [14–21]	15.5 [12.8–18]	0.011
RV S’, cm/s	11 [8.93–13]	8 [7–9.6]	0.004
TR velocity, m/s	2.9 [2.4–3.4]	3 [2.6–3.7]	0.086
RV–RA gradient, mmHg	30 [22–39]	34.5 [27–42]	0.027
Estimated PASP, mmHg	39 [28–52]	46.6 [36.4–63.8]	0.027
TAPSE/PASP	0.46 [0.28–0.65]	0.32 [0.22–0.47]	0.006
IVC diameter, mm	18 [14–22]	20 [17.8–24]	0.029

TAPSE, tricuspid annular plane systolic excursion; RV S’, peak systolic tricuspid annular velocity measured by tissue Doppler imaging; TR velocity, tricuspid regurgitation; RV–RA gradient, right ventricle–right atrium gradient; PASP, pulmonary artery systolic pressure; IVC diameter, inferior vena cava diameter.

**Table 8:** LVEF according to phenotypes and in-hospital outcome.

Phenotype	LVEF	Survivors	Non-Survivors	*p*-Value
Non-congested/preserved perfusion	53 [40–60]	54 [42–60]	42 [29.3–52.5]	0.235
Congested/preserved perfusion	44 [31.5–55]	42.5 [31.3–55]	45 [33–55]	0.335
Non-congested/impaired perfusion	46 [29–55]	46.5 [31–55]	29 [29–29]	0.430
Congested/impaired perfusion	38 [27–50]	39 [28–50]	35 [20–50]	0.236

Data are presented as median [IQR].

**Table 9:** Acute treatment according to congestion–perfusion phenotypes.

Acute Treatment	Non-Congested/Preserved Perfusion, *n* = 137	Non-Congested/Impaired Perfusion, *n* = 33	Congested/Preserved Perfusion, *n* = 415	Congested/Impaired Perfusion, *n* = 205	*p*-Value
Iv furosemide	78.1%	90.9%	97.3%	94.1%	<0.001
Inotropic support	7.3%	3.0%	9.2%	21.0%	<0.001
NIMV	2.2%	0.0%	14.2%	19.5%	<0.001
IMV	2.2%	3.0%	5.1%	10.7%	0.005

Data are presented as %. Iv, intravenous; NIMV, non-invasive mechanical ventilation; IMV, invasive mechanical ventilation.

## Data Availability

The data used in this study are available from the corresponding author upon request. The data are not publicly accessible due to patient privacy and confidentiality constraints.

## Abbreviations

The following abbreviations are used in this manuscript:

ADHF	Acute Decompensated Heart Failure
AHF	Acute Heart Failure
APE	Acute Pulmonary Edema
AUC	Area Under the Curve
CRP	C-reactive Protein
CI	Confidence Interval
DBP	Diastolic Blood Pressure
ED	Emergency Department
eGFR	Estimated Glomerular Filtration Rate
HF	Heart Failure
hs-cTnT	High-sensitivity cardiac Troponin T
IMV	Invasive Mechanical Ventilation
IQR	Interquartile range
Iv	Intravenous
IVC	Inferior Vena Cava
LV	Left Ventricle
LVEF	Left Ventricular Ejection Fraction
NIMV	Non-invasive Mechanical Ventilation
NT-proBNP	N-terminal pro-B-type Natriuretic Peptide
PASP	Pulmonary artery systolic pressure
RAP	Right Atrial Pressure
RV	Right Ventricle
RV–RA	Right Ventricular–Right Atrial
ROC	Receiver Operating Characteristic
S’	Doppler Tissue Imaging-derived tricuspid lateral annular systolic velocity
SBP	Systolic Blood Pressure
SPSS	Statistical Package for Social Sciences
TAPSE	Tricuspid Annular Plane Systolic Excursion
TR	Tricuspid Regurgitation
TRV	Tricuspid Regurgitation Velocity
